# Blunt Thoracic Trauma-Induced Mitral Papillary Muscle Avulsion with Pericardial Rupture and Cardiac Herniation: Difficult and Delayed Diagnoses

**DOI:** 10.1155/2020/3268253

**Published:** 2020-06-22

**Authors:** Ross McCauley, Faisal Shariff, Michael Steinberg, Thomas B. Bemenderfer, Patrick Davis, Mark Thompson, Christopher Lesh, Mark Walsh, Edward Evans

**Affiliations:** ^1^Indiana University School of Medicine, South Bend, IN 46617, USA; ^2^Memorial Hospital Trauma Center, 615 N. Michigan Street South Bend IN 46601, USA; ^3^Marian University College of Osteopathic Medicine, 3200 Cold Spring Road, Indianapolis, IN 46222, USA; ^4^St. Joseph Regional Medical Center, Mishawaka, IN 46545, USA

## Abstract

Blunt thoracic trauma (BTT) and the resultant isolated mitral papillary muscle avulsion, pericardial rupture, and cardiac herniation injuries are each rarely diagnosed clinical entities. We describe the first case of combined pericardial tear with cardiac herniation and ruptured mitral papillary muscles following BTT. Preoperative transesophageal echocardiography (TEE) diagnosed the delayed mitral papillary muscle rupture while all previous diagnostic modalities failed to delineate the pericardial rupture and cardiac herniation. Particular emphasis is placed on the clinical and radiologic aspects of the case that would heighten clinical suspicion in the emergency setting where blunt cardiac injury sequelae are suspected and frequently missed.

## 1. Introduction

Blunt thoracic trauma (BTT) is a primary cause of morbidity, mortality, hospitalization, and disability in the United States and is the major contributing factor in over a quarter of all trauma-related deaths [1, 2]. BTT can result in a variety of cardiac injuries, ranging from minor cardiac contusion to the more severe pathology such as cardiac rupture, pericardial rupture, cardiac herniation, valvular injury, myocardial infarction, or arrhythmia [1–3]. BTT-associated mitral valve injury, pericardial rupture, and cardiac herniation are independently very rare clinical entities with high mortalities and have not been reported together in the literature. We discuss the protean nature of the presentation of pericardial rupture with cardiac herniation where the diagnosis is often made retrospectively and intraoperatively. We present the literature's first case of combined mitral papillary muscle avulsion, pericardial rupture, and cardiac herniation and propose findings that, in retrospect, may expedite diagnosis.

## 2. Case Report

A 40-year-old female presented to the emergency department following a high-speed motor vehicle collision (MVC). Physical examination at the time of presentation to the emergency department revealed an alert and oriented patient with a pulse of 107 beats per minute, respirations of 30 per minute, blood pressure of 141/108 mmHg, and oxygen saturation of 92% on room air. The heart tones were of regular rhythm without murmurs, gallops, clicks, or rubs. There was no jugular venous distention or muffled heart tones. There was scattered wheezing noted bilaterally throughout the lung fields. Bruising was found on the left chest with significant tenderness to palpation but no crepitus or subcutaneous air. Pulses were palpable (2+) in all four extremities. Lacerations, bruising, and tenderness were found on various aspects of all four extremities. Motor control and sensation of extremities were grossly intact. EKG showed a normal sinus rhythm with no acute ST or T wave changes. Chest X-ray demonstrated mediastinal widening. Chest CT revealed left 2^nd^ through 7^th^ rib fractures anterolaterally, sternal fracture, mediastinal hematoma, a small left pneumothorax, grade III splenic laceration, and left pleural effusion. Transthoracic echocardiography was normal. Additional orthopedic injuries included a right intertrochanteric fracture, left pelvic acetabular fracture, and left talar neck fracture. Injuries are summarized in Table 1.

Three days after the MVC, the patient developed significant and rapidly worsening shortness of breath and bilateral opacification of lung fields on chest X-ray consistent with acute cardiogenic pulmonary edema. Vitals signs showed oxygen saturations in the mid-90s on Bi-Pap with 50% FiO_2_, respirations of 18 times per minute, and systolic blood pressure in the 90s. On auscultation of the thorax, there was a new 3/6 holosystolic heart murmur heard best at the apex with crackles two-thirds of the way up the lung fields bilaterally.

**Figure 1:**
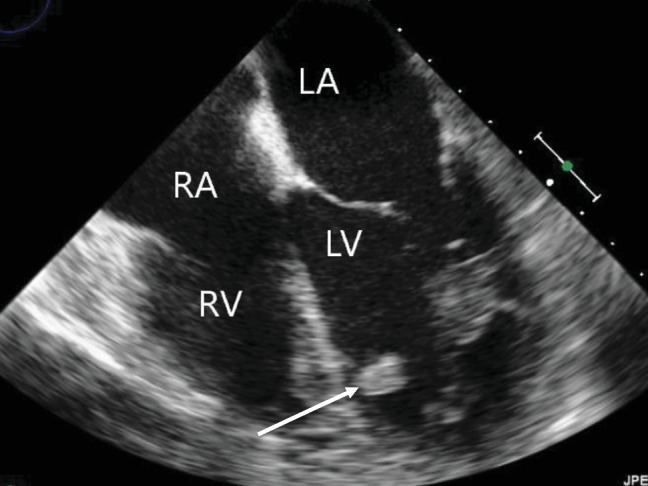
Transesophageal echocardiograph showing rupture mitral papillary muscle (arrow) in relation to the right atrium (RA), left atrium (LA), right ventricle (RV), and left ventricle (LV). TEE was obtained due to the presence of pulmonary edema with new holosystolic murmur on day 3 s/p MVC.

A repeat CT scan showed bilateral, diffuse alveolar air space disease confirming the diagnosis of pulmonary edema. There was no evident injury to the aorta. A transesophageal echocardiograph (TEE) demonstrated a normal-sized left atrium, severe 4+ mitral regurgitation, avulsion of the anteromedial mitral papillary muscle, normal ventricular function, and no evidence of pericardial effusion (Figure 1).

The patient underwent cardiac catheterization which confirmed the diagnosis based on the TEE of avulsion of the anteromedial mitral papillary muscle, and an intra-aortic balloon pump was placed.

The patient then underwent urgent sternotomy and open heart surgery for mitral valve replacement. Findings included (1) a full-thickness rupture of the pericardium from cardiac apex to the left atrium with pronounced cardiac herniation to the left, (2) anterior mitral papillary muscle was avulsed and located in a markedly abnormal position riding up towards the mitral valve apparatus almost sitting at the level of the annulus, (3) a large septal hematoma, and (4) a mid-sternal fracture. A number 25 Hancock II porcine tissue prosthesis was placed, and the pericardial tear repaired. The patient tolerated this procedure well, had no complications postoperatively, and was discharged to a rehabilitation facility where recovery was uneventful.

## 3. Discussion

Blunt thoracic trauma-induced mitral valve injury is exceedingly rare. In the classic pathologic study of blunt cardiac trauma, a series of 546 traumatic cardiac injuries found mitral valve injury in 0.01% of patients [4]. Due to the rarity of this clinical entity, there is little data on mortality following diagnosis and surgical repair. Pericardial rupture is more common, representing 0.3% of all multiple trauma [2], with mortality ranging from 30 to 64% in cases of pericardial rupture regardless of associated injuries [5, 6]. Cardiac herniation occurs in 0.4% of trauma and mortality ranges from 50% to 100% [2, 7]. The lethality of cardiac herniation is postulated to be caused by torsion and occlusion of the great vessels and is more common with leftward herniation of the heart [8].

As cardiac injury represents the most commonly unsuspected visceral injury responsible for death, it is critical to identify patients at risk for developing cardiac complications following BTT, especially the difficult to diagnose and often lethal pericardial rupture [9, 10]. Pericardial rupture and subsequent cardiac herniation frequently escape recognition because of their protean presentations. The failure of early diagnosis of these entities is due to the difficult detection of the subtle and evolving clinical, radiological, and electrocardiographic findings. The spectrum of signs and symptoms can vary greatly from subtle murmurs to fulminant cardiac decompensation or acute hemodynamic instability. These specific findings associated with pericardial rupture have been described previously, and our findings point to the diagnosis of pericardial rupture before it was discovered intraoperatively. The evolution of a harsh 3/6 holosystolic murmur that coincided with acute pulmonary edema caused the clinician to pursue the diagnosis of mitral papillary muscle avulsion and led to the intraoperative diagnosis of pericardial rupture and cardiac herniation [5, 10, 11]. The delay in this case is postulated to be due to the injury to the long penetrating arteries supplying the papillary muscles leading to ischemia and rupture [12, 13].

In addition, the diagnosis of such injuries can be significantly delayed secondary to the presence of other injuries. The diagnoses of these entities should be considered in BTT patients who have the following findings: hemodynamic instability, elevated jugular venous pressure, alternating heart tones, pulse, and blood pressures that change dramatically with body position shifts, characteristic murmur of mitral insufficiency, and water wheel or “bruit de moulin” like heart tones [5, 8, 10, 11, 14–16]. Although radiologic investigation is not sensitive, the literature indicates that certain findings on the chest X-ray, CT, and echocardiography are able to detect herniation [17–19]. Various radiological modalities, including contrast ultrasonography and CT have been proposed to be able to identify pericardial rupture, but no specific findings or algorithm has been described [9, 11, 20, 21]. We present this case as an example of the protean nature of BTT and three of its sequelae that are feared not only because of their high mortality but also difficult preoperative diagnoses. Fortunately, in this case, the mitral valve pathology prompted surgery that was able to discover and manage the pericardial rupture and cardiac herniation, two pathologies that almost certainly would have been lethal with further delay.

## 4. Conclusion

Pericardial rupture with cardiac herniation and mitral papillary muscle avulsion is rare, but highly lethal. This case demonstrates the need for trauma surgeons to recognize the limitations of traditional diagnostic modalities and the importance of heightened vigilance for suspecting cardiac and pericardial injury following significant BTT. Pericardial rupture and valvular injury may occur in patients presenting with BTT, even in the absence of traditionally suggestive clinical, physical, and radiological findings.

## Figures and Tables

**Table 1:** Patient injuries and mode of diagnosis.

Injury	Mode of diagnosis
Full thickness pericardial rupture from cardiac apex to left atrium	Sternotomy
Cardiac herniation	Sternotomy
Mediastinal hematoma	CT
Avulsion of anteromedial mitral papillary muscle	TTE, cardiac catheterization
Septal hematoma	Sternotomy
Fractures	CT, X-ray, physical exam
(i) Left 2nd-7th ribs	
(ii) Sternum	
(iii) Right humerus	
(iv) Right intertrochanteric	
(v) Left pelvic acetabular	
(vi) Left talar neck	
Grade III splenic laceration	CT
Left pneumothorax, pleural effusion	CT
Acute cardiogenic pulmonary edema 3 days s/p injury	CXR, CT

## References

[1] Lin J. C., Ott R. A. (1999). Acute traumatic mitral valve insufficiency. *The Journal of Trauma*.

[2] Fulda G., Brathwaite C. M. E., Rodriguez A., Turney S. Z., Dunham C. M., Cowley R. A. (1991). Blunt traumatic rupture of the heart and pericardium:. *The Journal of Trauma*.

[3] Julie Ottosen M. D. (2012). *WAGMPF. Blunt cardiac injury*.

[4] Parmley L. F., Manion W. C., Mattingly T. W. (1958). Nonpenetrating traumatic injury of the heart. *Circulation*.

[5] Wang H., Li M. (2013). Blunt traumatic pericardial Rupture&lt;br/&gt;—Case report and literature review. *Surgical Science*.

[6] Galindo Gallego M., Lopez-Cambra M. J., Fernandez-Acenero M. J. (1996). Traumatic rupture of the pericardium. Case report and literature review. *The Journal of Cardiovascular Surgery*.

[7] Veronesi G., Spaggiari L., Solli P. G., Pastorino U. (2001). Cardiac dislocation after extended pneumonectomy with pericardioplasty. *European Journal of Cardio-Thoracic Surgery*.

[8] Glotzer O. S., Bhakta A., Fabian T. (2014). Blunt force thoracic trauma: a case study of pericardial rupture and associated cardiac herniation. *Case Reports in Surgery*.

[9] Terry T. R., Cook A. (1983). Blunt injury of the heart. *BMJ*.

[10] Wall M. J., Mattox K. L., Wolf D. A. (2005). The cardiac pendulum: blunt rupture of the pericardium with strangulation of the heart. *The Journal of Trauma*.

[11] Schir F., Thony F., Chavanon O., Perez-Moreira I., Blin D., Coulomb M. (2001). Blunt traumatic rupture of the pericardium with cardiac herniation: two cases diagnosed using computed tomography. *European Radiology*.

[12] Shaikh N., Ummunissa F., Abdel S. M. (2013). Traumatic mitral valve and pericardial injury. *Case Reports in Critical Care*.

[13] Pasquier M., Sierro C., Yersin B., Delay D., Carron P. N. (2010). Traumatic mitral valve injury after blunt chest trauma: a case report and review of the literature. *Journal of Trauma and Acute Care Surgery*.

[14] Sherren P. B., Galloway R., Healy M. (2009). Blunt traumatic pericardial rupture and cardiac herniation with a penetrating twist: two case reports. *Scandinavian Journal of Trauma, Resuscitation and Emergency Medicine*.

[15] Rippey J. C. R., Rao S., Fatovich D. (2004). Blunt traumatic rupture of the pericardium with cardiac herniation. *CJEM*.

[16] Crynes S. F., Hunter W. C. (1939). Traumatic rupture of the pericardium. *Archives of Internal Medicine*.

[17] Matsuda S., Hatta T., Kurisu S., Ohyabu H., Koyama T., Kita Y. (1999). Traumatic cardiac herniation diagnosed by echocardiography and chest CT scanning: report of a case. *Surgery Today*.

[18] Carrillo E. H., Heniford B. T., Dykes J. R., McKenzie E. D., Polk H. C., Richardson J. D. (1997). Cardiac herniation producing Tamponade. *The Journal of Trauma*.

[19] Cook F., Mounier R., Martin M., Dhonneur G. (2017). Late diagnosis of post-traumatic ruptured pericardium with cardiac herniation. *Canadian Journal of Anaesthesia*.

[20] Tatekoshi Y., Yuda S., Ogasawara M. (2016). Successful diagnosis of pericardial rupture caused by blunt chest trauma using contrast ultrasonography. *Journal of Medical Ultrasonics*.

[21] Kirsch J. D., Escarous A. (1989). CT diagnosis of traumatic pericardium rupture. *Journal of Computer Assisted Tomography*.

